# SMArtCARE - A platform to collect real-life outcome data of patients with spinal muscular atrophy

**DOI:** 10.1186/s13023-019-0998-4

**Published:** 2019-01-21

**Authors:** Astrid Pechmann, Kirsten König, Günther Bernert, Kristina Schachtrup, Ulrike Schara, David Schorling, Inge Schwersenz, Sabine Stein, Adrian Tassoni, Sibylle Vogt, Maggie C. Walter, Hanns Lochmüller, Janbernd Kirschner

**Affiliations:** 1 Department of Neuropediatrics and Muscle Disorders, Medical Center- University of Freiburg, Faculty of Medicine, University of Freiburg, Freiburg, Germany; 2 Clinical Trials Unit, Medical Center – University of Freiburg, Faculty of Medicine, University of Freiburg, Freiburg, Germany; 3 Gottfried von Preyer Children Hospital, Vienna, Austria; 4 Department of Neuropediatrics and Neuromuscular Centre for Children and Adolescents, Children’s Hospital 1, University of Duisburg-Essen, Essen, Germany; 5 Deutsche Gesellschaft für Muskelkranke e. V., Freiburg, Germany; 6 Department of Physiotherapy, Albert-Ludwigs University of Freiburg, Medical Center, Freiburg, Germany; 7 Department of Neurology, Friedrich-Baur-Institute, Ludwig-Maximilians-University of Munich, Munich, Germany; 8 Division of Neurology, Department of Medicine, The Ottawa Hospital Ottawa Children’s, Canada and Hospital of Eastern Ontario Research Institute, University of Ottawa, Ottawa, Canada

**Keywords:** Spinal muscular atrophy, Real-life outcome data, Orphan drug, Rare disease, Registry

## Abstract

**Background:**

Survival and quality of life for patients affected by spinal muscular atrophy (SMA) are thought to have improved over the last decade due to changes in care. In addition, targeted treatments for SMA have been developed based on a better understanding of the molecular pathology. In 2016 and 2017, nusinersen was the first drug to be approved for treatment of all types of SMA in the United States and in Europe based on well-controlled clinical trials in a small subgroup of pediatric SMA patients. Systems are required to monitor treated and untreated SMA patients in a real-life environment to optimize treatment and care, and to provide outcome data to regulators, payers, and the SMA community.

**Methods:**

Within SMArtCARE, we conduct a prospective, multicenter non-randomized registration and outcome study. SMArtCARE collects longitudinal data on all available SMA patients independent of their actual treatment regime as disease-specific SMA registry. For this purpose, we provide an online platform for SMA patients seen by health-care providers in Germany, Austria and Switzerland. All data are collected during routine patient visits. Items for data collection are aligned with the international consensus for SMA registries. Data analysis is carried out independent of commercial partners.

**Conclusion:**

A prospective monitoring of all SMA patients will lead to a better understanding of the natural history of SMA and the influence of drug treatment. This is crucial to improve the care of SMA patients. Further, we will establish a network for neuromuscular centers to share experience with SMA patients and to promote research projects on SMA.

**Trial registration:**

German Clinical Trials Register (“Deutsches Register klinischer Studien”) DRKS00012699. Registered 09 August 2018. https://www.drks.de/drks_web/navigate.do?navigationId=trial.HTML&TRIAL_ID=DRKS00012699.

**Electronic supplementary material:**

The online version of this article (10.1186/s13023-019-0998-4) contains supplementary material, which is available to authorized users.

## Background

Spinal muscular atrophy (SMA) is an autosomal-recessive disorder caused by a homozygous deletion in the survival motor neuron (*SMN*) 1 gene on chromosome 5q13. With an incidence of 1:10,000, SMA is classified as a rare disease [1]. SMA is characterized by degeneration of the anterior horn cells of the spinal cord resulting in muscle atrophy and proximal muscle weakness. Depending on the severity of symptoms and age of onset, SMA was divided into five different subtypes, although it is now apparent that the phenotype of SMA rather spans a broad continuum without a clear delineation of subtypes. From the time point of diagnosis, patients with SMA rarely achieve improvements of motor function or acquire additional motor developmental milestones.

The natural history of SMA has changed over the last decade due to improvements in care. For example, with non-invasive ventilator support and enteral feeding, survival of the critically ill infants with SMA type 1 has increased [2, 3]. A revised consensus statement for standards of care in SMA has been published recently [4, 5], but nonetheless, there is high variability in the care for SMA patients.

Targeted treatments for SMA have been developed in preclinical and clinical studies. Among these, several drugs have already reached clinical trials of phase I-III [6–8]. Nusinersen was the first drug to be approved for treatment of all types of SMA based on well-controlled clinical trial data of a small subgroup of pediatric SMA patients [9, 10]. Prior to approval in Europe, nusinersen was provided to patients with SMA type 1 within an Expanded Access Program (EAP). Data of all available patients were systematically collected in 7 neuromuscular centers in Germany so that we could evaluate the response to treatment with nusinersen in a broader spectrum of SMA type 1 patients [11]. But still, considering current age and disease stage, most SMA patients are outside the population that has been studied in randomized controlled trials. Thus, there is a strong need to monitor all treated and untreated SMA patients in a real-life environment to optimize treatment and care.

The aim of the SMArtCARE project is to collect longitudinal data on all available SMA patients independent of their actual treatment regime as disease-specific SMA registry. A prospective monitoring of SMA patients is expected to lead to a better understanding of the natural history of SMA and the influence of drug treatments. In the following, we will describe the design and set-up of the SMArtCARE project as a platform to collect real-life outcome data of patients with SMA.

## Methods

### Study design

We conduct a prospective, multi-center non-randomized registration and outcome study in Germany, Austria and Switzerland. For this purpose, we provide an online platform for SMA patients seen by health-care providers. This data collection is conducted in addition to the existing SMA patient registry in Germany (www.sma-register.de).

**Fig. 1:**
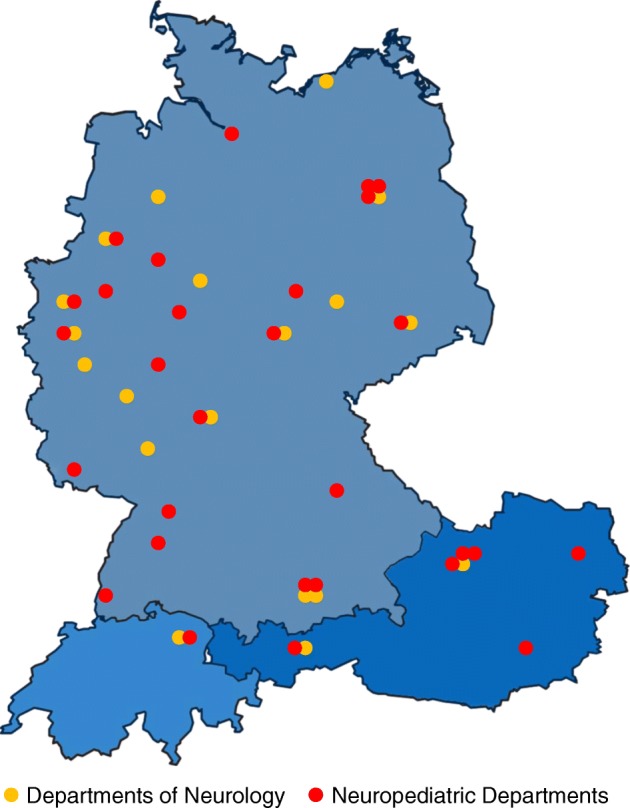
Participating centers in Germany, Austria and Switzerland (status 11/2018)

All neuromuscular centers that care for patients with SMA are invited to participate in this study. Neuromuscular centers that provide treatment with nusinersen were identified by the German Society for people with Muscular Diseases (Deutsche Gesellschaft für Muskel-kranke (DGM) e.V.). To date, already 50 neuromuscular centers confirmed their participation in the SMArtCARE data collection so that we expect data of approximately 1,000 SMA patients within the first year of the study. The participating centers are displayed in Fig. 1.

Inclusion criteria for patients are defined as genetically confirmed 5q-SMA and that the patient or the caregivers have given a written informed consent. To avoid selection bias, physicians are asked to include all patients fulfilling the inclusion criteria in a consecutive way independent on their treatment regime.

The SMArtCARE steering committee includes representatives of child neurologists (Janbernd Kirschner (Freiburg), Ulrike Schara (Essen), Günther Bernert (Vienna)) and neurologists (Maggie C. Walter (Munich), Hanns Lochmüller (Freiburg)) from Germany and Austria and a representative of the SMA patient organization (Inge Schwersenz (Initiative SMA)). The main responsibilities of the Steering Committee are to supervise the project progress, to approve amendments and research proposals and to create reports for participating centers and sponsors.

### Data collection

Items for data collection are aligned with the international consensus for SMA registries (TREAT-NMD, iSMAC). Data are collected during routine patient visits. The timing and frequency of follow-up visits and assessments depend on the actual treatment regime. SMA patients are normally seen once or twice a year in a specialized outpatient clinic for neuromuscular diseases. Under treatment with nusinersen, for example, follow-up visits are scheduled every four months. Recommendations for the clinical evaluation of SMA patients are summarized in Table 1.

**Table 1:** Recommendations for the evaluation of patients with SMA

	Patient visits (days)					
	1	14^a^	28^a^	63^a^	180	Every 6 months (every 4 months^a^)
**Baseline data** including genetic test results	**X**					
**Current medical history and clinical examination** - including motor milestones in children < 12 years of age	**X**	**X**	**X**	**X**	**X**	**X**
**Administration of nusinersen** ^a^	**X**	**X**	**X**	**X**	**X**	**X**
**Physiotherapeutic assessments:** **CHOP INTEND** • All children < 2 years of age • All patients > 2 years of age without ability to sit **HFMSE** • All patients > 2 years of age with ability to sit • If CHOP INTEND score > 50: CHOP INTEND and HFMSE • If CHOP INTEND score > 60: HFMSE instead of CHOP INTEND **RULM** • All patients > 2 years of age with ability to sit (in a wheelchair) **6-MWT** • All ambulant patients > 3 years of age	**X**			**X**	**X**	**X**
**ALS Functional Rating Scale** (in adult patients)	**X**			**X**	**X**	**X**
**Pulmonary function** ^b^	**X**				**X**	**X**
**Documentation of adverse events**	**Ongoing**					

^a^Additional recommendations under treatment with nusinersen

^b^If the patient is sufficiently cooperative due to age

As baseline characteristics, we ask for data regarding sex, age at onset of symptoms, genetic test results including *SMN2* copy number, motor function, use of ventilator support, nutritional status, orthopedic symptoms, pre-existing illnesses, additional therapies, concomitant medication and the family medical history. During every patient visit current data regarding respiratory and nutritional status, orthopedic symptoms including experience of pain and fatigue are evaluated among other things. Adverse events and the subjective impression of the patient or caregiver are documented. In pediatric patients, growth parameters are recorded during every patient visit. Further, a clinical examination including an assessment of contractures and the current motor function is performed. In children under 12 years of age, the Hammersmith Infant Neurological Examination (HINE) [12] is used to evaluate motor milestones. In patients treated with nusinersen, further data regarding the procedure of lumbar punctures, the need for an additional sedation or general anesthesia, and adverse events during lumbar punctures are collected. A table with all data elements is provided in the supplement (see additional file 1).

Several functional motor scales were developed for patients with SMA as clinical tools but as well as functional outcome measures for clinical trials. For the SMArtCARE database, we use the Children’s Hospital of Philadelphia Infant Test of Neuromuscular Disorders (CHOP INTEND) [13] in children under 2 years of age and in patients without ability to sit independently. The Hammersmith Functional Motor Scale Expanded (HFMSE) [14] is performed in children older than 2 years of age with ability to sit and in patients with a CHOP INTEND score > 50. To evaluate motor function of upper limbs, we recommend the Revised Upper Limb Module (RULM) [15] in patients older than 2 years of age with the ability to sit in a wheelchair. In ambulant patients older than 3 years of age, the 6-Minute-Walk Test (6MWT) [16] is performed additionally. Recommendations for the physiotherapeutic assessments are summarized in Table 1. Two-day central training workshops are organized for participating physiotherapists.

In adult patients the revised Amyotrophic Lateral Sclerosis (ALS) functional rating scale [17] is used to explore the progress of the disease. Further, pulmonary function tests are performed to evaluate forced vital capacity and peak cough flow.

Patient reported outcome measures are an important tool to evaluate quality of life of SMA patients. Also to further investigate symptoms as pain, fatigue or swallowing difficulties, patient reported outcomes are crucial. Validated SMA-specific questionnaires are still very sparse, so that questionnaires for patients with neuromuscular diseases or generic quality of life questionnaires have to be used. Especially for more severely affected patients with SMA there is a need to develop new outcome measures to reliably measure functions such as swallowing, pain, fatigue, and residual (minimal) motor function. The SMArtCARE data collection will enable to perform validation studies for these novel outcome measures.

### IT platform

For the SMArtCARE data collection, an electronic data capture (EDC) system is used. This system is a web-based data entry system that is developed by OpenApp LTD and hosted within the IT infrastructure of the University Medical Center Freiburg. For data entry, different, standardized e-forms are provided. The aim of the IT platform is to support care of SMA patients with a standardized anamnesis questionnaire in accordance with the published standards of care [4, 5]. To facilitate the management and care of SMA patients, specific features are implemented in the IT system. Based on the age of the patient and the previously entered data, the respective selection of e-forms is proposed when planning a new patient visit. The next visit is scheduled dependent on the current treatment of the patient. Reports can be printed for every patient visit to reduce the documentation effort. For each patient, a diagram is displayed to give an overview of changes in motor or respiratory function, adverse events or drug treatment for the investigator. Further, to report drug-related adverse events, the system will provide a printable, standardized form that can be transferred to the respective authorities. If sub-studies are conducted using the infrastructure of SMArtCARE, specific e-forms can be implemented in the IT system and, if required, these additional e-forms can be used only in a sub-group of patients.

### Research network

SMArtCARE aims to establish a research network among neuromuscular centers in German-speaking countries to share experience with SMA patients and to collaborate in research projects. The infrastructure of the SMArtCARE database, as well as the dataset, can be used for different research projects. The SMArtCARE steering committee will decide on the implementation of research projects based on a written synopsis of the respective neuromuscular center.

### Ethical considerations

Ethics approval of the central ethics committee has been obtained (EK 56/18). The study is registered in the German Clinical Trials Register (“Deutsches Register klinischer Studien” (DRKS00012699)). Patients are asked for their informed consent prior to any data entry into the online system. Consent forms are provided as supplemental material (see Additional files 2, 3, 4 and 5). The procedure of patients' informed consent is regulated according to data protection laws, in particular the General Data Protection Regulation (GDPR). In the case of a withdrawal of a patient’s consent, all data is anonymized immediately and, if requested, all data is deleted completely. For research projects data will generally be distributed as anonymized data batches. When entering a new patient, a patient identifier code is generated to pseudonymize the patient identifying data. This identifier code is unique to each patient and retains as primary identifier for the patient throughout the entire participation in the registry. Only the treating center is able to see the patient identifying data. This code prevents duplicate entries of patients within the SMArtCARE database.

To avoid multiple registrations of a single patient in different registries and to share data, there are different approaches to establish a Privacy-Preserving Report Linkage (PPRL). A PPRL is a unique identifier to create a research dataset in which all records pertaining to the same person are linkable [18]. Within the SMArtCARE database, we will collect all identifying data to allow generating a PPRL.

### Data quality

SAS software is used to review data for completeness, consistency and plausibility. For certain items in the data set, only reasonable values can be entered to avoid typing errors. We defined a selection of mandatory items to ensure a minimal data set of all patients. Data quality will be assessed by describing recruitment figures and data completeness (percentages of missing values). Categorical data will be summarized by the total number of patients in each category and the number of missing values. Relative frequencies are displayed as valid percentages.

### Financing

Biogen provides initial financial support for this registry. Sponsor of the study is the University Medical Center Freiburg. The funding source had no influence on the design and data elements of this registry. The academic SMArtCARE network owns the data sovereignty with all responsibilities for data protection, data analysis and the right to publish results of the study. Additional financial support from other companies is intended to secure long-term sustainability of the registry.

## Discussion

SMArtCARE aims to collect longitudinal data on all available SMA patients as disease-specific SMA registry. Compared to drugs in common diseases, approval of orphan drugs is often based on a limited amount of evidence. After approval it is often not feasible to conduct further placebo-controlled clinical trials. But to evaluate the long-term effect of these drugs in a broad spectrum of patients, it is crucial to collect clinical data on the respective patients systematically and independent of commercial partners. For the treatment of all types of SMA, nusinersen is the first drug to be approved, but further drugs have already reached clinical trials of phase I-III [6–8]. Therefore, the aim of the SMArtCARE registry is to include all SMA patients independent of their current treatment regime. Disease-specific registries are favored by all stakeholders over product-specific registries to allow meaningful analysis across both treated and untreated SMA patients [19]. A comprehensive collection of post-marketing data is challenging not only due to paucity of data but also due to the lack of options to use the existing data. There is an international consensus on the need of data sharing and the FAIR principles of data (findable, accessible, interoperable, reusable) [19]. Therefore, the interoperability of different approaches establishing SMA registries to collect real-life outcome data throughout the world is crucial. Within SMArtCARE, items for data collection are aligned with the international consensus for SMA registries [1, 19, 20]. For the evaluation of patients with SMA, we developed a standardized data set in accordance with other international SMA registries such as TREAT-NMD and iSMAC and used proved ontologies, if applicable. Further, there is a strong effort to identify biomarkers or genetic modifiers to better understand the high variability of phenotype in SMA patients and also to investigate the effects of drug treatment in different subgroups of SMA patients. Here, clinical data are necessary to evaluate the correlation between biomarkers and phenotype.

## Conclusion

The evaluation of real-life outcome data in a broad spectrum of SMA patients will lead to a better understanding of the natural history and the influence of drug treatment. This is crucial to revise and implement standards of care for these patients. The IT system of the SMArtCARE database will help to implement recommendations for the care of SMA patients according to the international consensus for standards of care [4, 5]. To enable an international collaboration and data sharing, data items are standardized and aligned with the international consensus of SMA registries. At last, we strive to establish a network for neuromuscular centers in Germany, Austria and Switzerland to share experience with SMA patients and to promote research projects using the infrastructure of the SMArtCARE database.

## Additional files

Additional file 1: Data items SMArtCARE. Table with all data elements of the SMArtCARE platform. (PDF 319 kb)

Additional file 2: SMArtCARE patient information sheet for children aged 7–11 years. Consent form for the SMArtCARE data base for children aged 7–11 years. (PDF 78 kb)

Additional file 3: SMArtCARE patient information sheet for children aged 12–16 years. Consent form for the SMArtCARE data base for children aged 12–16 years. (PDF 74 kb)

Additional file 4: SMArtCARE parent information sheet. Consent form for the SMArtCARE data base for parents. (PDF 106 kb)

Additional file 5: SMArtCARE patient information sheet. Consent form for the SMArtCARE data base for patients. (PDF 104 kb)
