# Supplementary material for: SMArtCARE - A platform to collect real-life outcome data of patients with spinal muscular atrophy
Source: Orphanet J Rare Dis. 2019 Jan 21;14:18. doi: 10.1186/s13023-019-0998-4 (PMC6341722; doi:10.1186/s13023-019-0998-4)
Supplement: Supplementary file 1 — Data items SMArtCARE. Table with all data elements of the SMArtCARE platform. (PDF 319 kb) [file 13023_2019_998_MOESM1_ESM.pdf]

Data items SMArtCARE

	Data items	Answers	If yes:		Comments

Enrolment

**Enrolment**	Date enrolled				automatically generated
	Date of consent				
	Genetically proven 5q SMA				
	First name				
	Last name				
	Last name at birth				
	Date of birth				
	Country of birth				
	City of birth				
	Country of residence	Austria<br>Germany<br>Switzerland<br>Other (specify)			
	Patient identification number				internal hospital patient ID
	Email address				
	Gender	Male<br>Female<br>Undifferentiated<br>Unknown			

Baseline

**Genetic Test Result**	Do you have a copy of the genetic report?	Yes<br>No	Date of genetic report		
			Name of genetic institute		
			Type of mutation	Homozygous deletion involving exon 7 of SMN1<br>Heterozygous deletion and heterozygous point mutation (compound heterozygotes)<br>Other (specify)	
	SMN2 copy number performed?	Yes<br>No	*SMN2* copy number	1, 2, 3, 4, 5, 6, 7, 8<br>other (specify)	
			Molecular testing approach of *SMN2*	MLPA<br>Sequence analysis<br>Other (specify)	
	Was diagnosis made pre-symptomatically?	Yes<br>No	Screening type	Newborn screening<br>Positive family history<br>Other (specify)	
	Any other family member affected?	Yes<br>No<br>Unknown			Kinship as auto-complete
**Clinical diagnosis**	First symptoms or signs leading to suspect SMA. Please specify.				
	Age at symptom onset	Prenatal<br>At birth<br>Within the first 4 weeks<br>At the age of (specify)			
	Sitting without support<br>Crawl on hands and knees<br>Standing without support<br>Walking without	Never able<br>Gained<br>Gained and lost	Age gained<br>Age lost		Crawl on hands on knees only in patients < 18 years of age

	support<br>Climb stairs				
	Wheelchair use?	No<br>Part-time<br>Full-time	Start date of wheelchair use		Wheelchair use only in patients > 2 years of age
	Does the patient receive ventilator support?	Yes<br>No	Type of ventilator support	Non-invasive<br>Invasive	
				Start date of ventilator support	
	Does the patient use a gastric or nasal feeding tube?	Yes – supplementary e.g. for fluids<br>Yes - exclusively fed by tube<br>No	Start date of tube feeding		
	Scoliosis surgery?	Yes<br>No	Date of surgery		
			Surgery technique	Arthrodesis<br>Growing rods<br>Other (specify)	
	Any other pre-existing illness?	Yes<br>No<br>Unknown	Add pre-existing illness		ICD 10 code
**Registries, clinical trials**	Is the patient currently or was previously included in a clinical trial?	Yes<br>No	Currently included<br>Previously included		
			Name of trial		
			Name of drug		
			Start date		
			End date		
	Is the patient currently enrolled in other registries?	Yes<br>No<br>Unknown	Specify		

Medical Assessment

	Visit date				
	Age at visit				automatically filled, years and months
	Physician's first name				
	Physician's last name				
**Pulmonary**	Does the patient receive ventilator support?	Yes<br>No	Type of ventilation	Non-invasive<br>Invasive	
				Date of change	
			Frequency of use	Daily use<br>Occasional use<br>Use when ill only	
			Time of ventilator use	Night (during sleep)<br>Intermittent day time and continuous at night<br>Continuous (>16h/day)<br>Intermittent with acute illnesses	
			Hours per day	1-24	
			Start of ventilator use (date)<br>Ongoing<br>End of ventilator use (date)		
	Assistance in airway clearance and secretion mobilisation	Yes<br>No	Type of airway clearance and secretion mobilisation	Suction<br>Chest physiotherapy<br>Cough assist device<br>Other (specify)	
			Frequency of use	Daily use<br>Occasional use<br>Use when ill only	
	Pulmonary function performed?	Yes<br>No	Date of pulmonary function		
			FVC percentage		

			FVC litre		
			Peak cough flow		
	Vaccination performed?	Yes<br>No	RSV	Date of last vaccination	
			Influenza	Date of vaccination	
			Pneumococcus	Date of vaccination	
**Nutrition**	Does the patient use a gastric or nasal feeding tube?	No<br>Yes - exclusively fed by tube<br>Yes – supplementary e.g. for fluids	Start of tube feeding (date)<br>Ongoing<br>End of tube feeding (date)		
	Swallowing?	Normal<br>With difficulties			
	Chewing?	Normal<br>With difficulties			Only in patients > 1 year of age
**Orthopaedics**	Does the patient have scoliosis?	Yes<br>No	Was new x-ray performed?	Yes<br>No	
			Date of x-ray		
			Cobb angle		
	Orthopaedic surgery since last visit?	Yes<br>No	Type of orthopaedic surgery	Scoliosis surgery<br>Other types of orthopaedic surgery (specify)	
			Surgery technique	Arthrodesis<br>Growing rods<br>Other (specify)	
	Does the patient suffer pain regularly?	Yes<br>No<br>Unknown	Pain intensity	Mild<br>Moderate<br>Severe	
			Localisation		
	Does the patient experience fatigue?	Yes<br>No<br>Unknown	Specify		
**Hospitalisation**	Planned hospitalisation since last visit (except for	Yes<br>No<br>Unknown	Admission date<br>Ongoing<br>Discharge date		

	treatment administration)?				
			Reason for hospitalisation	Sleep study<br>Placement of gastric tube<br>Orthopaedic surgery<br>Other (specify)	
**Therapy**	Therapy interventions?	Physiotherapy<br>Feeding / Speech<br>Occupational Therapy<br>Other (specify)			
	Orthoses?	Yes<br>No	Type of orthoses	Ankle-foot Orthoses (AFOs)<br>Supramalleolar Orthoses (SMO)<br>Orthotics/shoe inserts<br>Knee-ankle-foot Orthoses (KAFOs)<br>Hand splints<br>Other (specify)	
			Orthotic Type of Use	Functional, Day time only<br>Resting Day time<br>Resting Night time	
			Frequency (times per week)	1-7	
	Devices?	Yes<br>No	Type of devices	Standing Frame<br>Positioning Device (Seating system)<br>Therapy bicycle<br>Galileo<br>Other (specify)	
			Frequency (times per week)	1-7	
	Wheelchair use?	No<br>Part-time<br>Full-time	Type of wheelchair	Manual wheelchair<br>Power wheelchair	Only in patients > 2 years of age
			Start date		
**Medication**	Is the patient on any	Yes	Name of drug		

	approved medication for SMA	No			
			Start date<br>Ongoing<br>End date		
	Other medication taken on a regular basis	Yes<br>No	Name of medication		
			Start date<br>Ongoing<br>End date		
**Clinical Trial**	Is the patient currently included in a clinical trial?	Yes<br>No<br>Unknown	Name of trial		
			Name of drug		
			Start date		
**Best current motor function**	Any changes in motor milestones?	No<br>Improvement<br>Worsening	Best current motor function	Sitting without support<br>Crawl on hands and knees<br>Standing without support<br>Walking without support<br>Climb stairs<br>None of the above	Crawl on hands and knees only in patients < 18 years of age
			Age gained of new motor milestone		
			Age loss of previous motor milestone		
**Upper limb function**	Achieve useful function of hands (e.g. hold a pen or pick pennies up from a table)	Yes<br>No			
	Raise hands to mouth (in a sitting position)	Yes<br>No			
	Reach overhead (in a sitting position)	Yes<br>No			

**HINE**					In patients < 12 years of age
**Clinical examination**	Height (cm)				Percentile auto-calculated in patients < 18 years of age
	Body weight (kg)				Percentile auto-calculated in patients < 18 years of age
	Head circumfereance (cm)				Percentile auto-calculated in patients < 3 years of age
	BMI				Auto-calculated
	Skin	Normal<br>Abnormal<br>Not examined	Specify		
	HEENT	Normal<br>Abnormal<br>Not examined	Specify		
	Pulmo	Normal<br>Abnormal<br>Not examined	Specify		
	Heart	Normal<br>Abnormal<br>Not examined	Specify		
	Abdomen	Normal<br>Abnormal<br>Not examined	Specify		
	Neurology	Symptoms related to SMA<br>Neurological findings not related to SMA<br>Not examined	Specify		
	Are any contractures present?	Yes<br>No	Type of limitation	Minimal (no limitations to function)<br>Moderate (some limitations, adaptions necessary)<br>Severe (imposing limits to function)	
			Description of contractures / localisation		

**Laboratory**	Laboratory testing performed?	Yes<br>No	Any abnormalities of clinical significance?	Yes (specify)<br>No	
**Neuro-physiology** (optional)	Motor nerve conduction velocity (m/s)	Ulnar<br>Median<br>Tibialis			
	F-wave latency (ms)	Ulnar<br>Median<br>Tibialis			
	CMAP amplitude (mV)	Ulnar<br>Median<br>Tibialis			
**PROM**	General impression	Much improved<br>Minimally improved<br>No changes<br>Minimally worse<br>Much worse	Specify		In patients > 12 years of age: description of the impression in comparison to 12 months ago.<br>In patients <=12 years of age: description of the impression in comparison to 6 months ago.<br>In patients < 12 months of age: description of the impression in comparison to 3 months ago.
	Motor function	Much improved<br>Minimally improved<br>No changes<br>Minimally worse<br>Much worse	Specify		
	Respiratory function	Much improved<br>Minimally improved<br>No changes<br>Minimally worse<br>Much worse	Specify		

Adverse Events

**Adverse events**	Date recorded				automatically generated
	Has there been any adverse event since the last visit?	Yes<br>No			
	Has there been unplanned or prolonged hospitalisation?	Yes<br>No	Type of unexpected event	Respiratory tract infection<br>Hydrocephalus<br>Epileptic seizure<br>Post lumbar puncture syndrome<br>Other (specify)	
			Medra code of acute event		
			Description of acute event		
			Admission date<br>Ongoing<br>Discharge date		
			Drug treatment for post lumbar puncture syndrome necessary?	Yes<br>No<br>Unknown	
			Type of drug treatment for post lumbar puncture syndrome	Analgesics<br>Caffeine<br>Other (specify)	
			Is the adverse event related to drug treatment?	No<br>Possibly<br>Yes	If “possibly” or “yes”: Generation of a standardized form to report drug related adverse events to the respective authority
			Name of drug		
	Any unexpected events without hospitalisation?	Yes<br>No	Type of unexpected event	Respiratory tract infection<br>Hydrocephalus<br>Epileptic seizure<br>Post lumbar puncture syndrome<br>Other (specify)	

			Medra code of acute event		
			Description of acute event		
			Start date<br>Ongoing<br>End date		
			Post lumbar puncture syndrome?	Drug treatment for post lumbar puncture syndrome necessary?	
			Type of drug treatment for post lumbar puncture syndrome	Analgesics<br>Caffeine<br>Other (specify)	
			Is the adverse event related to drug treatment?	No<br>Possibly<br>Yes	If “possibly” or “yes”: Generation of a standardized form to report drug related adverse events to the respective authority
			Name of drug		

Nusinersen

**Nusinersen**	Date of treatment				
	Care Setting	Outpatient<br>Inpatient (overnight)			
	Ventilator support during LP	Yes<br>No	Type of ventilator support	General anaesthesia<br>Patient's ventilator device	
	Anaesthesia or sedation	Yes<br>No	Drug		
			Application	Intranasal<br>Intravenous<br>Peroral<br>Other (specify)	
	Use of	Ultrasound<br>Fluoroscopy/radioscopy<br>CT-Scan	Radiation dose of CT scan		

		Other (specify)			
	Duration of procedure				
	Adverse events during procedure	Yes<br>No	Specify		

End of data collection

**End of data collection**	Date recorded				automatically generated
	Has the patient withdrawn the consent?	Yes<br>No			
	Is the patient deceased?	Yes<br>No	Date of death		
			Cause of death		ICD 10 code
	Other reason for end of data collection				
