# Supplementary material for: SMArtCARE - A platform to collect real-life outcome data of patients with spinal muscular atrophy
Source: Orphanet J Rare Dis. 2019 Jan 21;14:18. doi: 10.1186/s13023-019-0998-4 (PMC6341722; doi:10.1186/s13023-019-0998-4)
Supplement: Supplementary file 2 — SMArtCARE patient information sheet for children aged 7–11 years. Consent form for the SMArtCARE data base for children aged 7–11 years. (PDF 78 kb) [file 13023_2019_998_MOESM2_ESM.pdf]

Space for patient label

SMArtCARE
Contact person:

PATIENT INFORMATION SHEET
For children aged 7-11 years
Data collection on disease progression in patients with spinal muscular atrophy

Project short title: SMArtCARE

Dear ______________

You regularly attend our clinic because you are not well. To find out how best to help you and other children with your disease, we would like to collect all the information to do with your disease.

For this reason, we have asked your parents to let you take part in the data collection project described below. On the next few pages, we would like to explain what this data collection project involves and what taking part will mean for you. You can ask us questions at any time, which we will be happy to answer.

What will this data collection project involve?

As you have already learned from your doctor or parents, you have a disease known as spinal muscular atrophy (SMA, for short). This disease causes muscle weakness, so that you do not always find it easy to move and you are unable to move as you would like. It can sometimes be difficult for you to breathe or cough, so you have trouble fighting off chest infections. Weakness of the muscles can cause a spine deformity known as scoliosis.

It is not yet clear exactly how best to help children with your disease. There are already various options available, but we don't really yet know which one works best. For this reason, we are collecting data in this registry, so that we can better understand what may help children with your disease.

Apart from yourself, up to 1,000 patients in German-speaking countries are to take part in this data collection project. As you have an extremely rare disease, similar data collection projects are currently being set up in various countries throughout Europe. Doctors are also working together on this internationally to exchange data, in order to get a better scientific understanding on the progression of the disease. Of course, your personal data will remain secure, so that no-one will be able to link the data back to you.

What will happen in this data collection project?

If you take part in this data collection project, you will continue to be treated as normal and your doctor will examine you in exactly the same way as during your previous visits to the doctor. Regarding the examination, it will make no difference whether or not you take part in the data collection project. As well as being examined, you will regularly be asked questions about your well-being. This will also be the same as at your previous visits to your doctor. In addition, our physiotherapists will carry out some tests on you to see how well you can move.

If you and your parents agree to take part in the project, your data will be entered into a database. To begin with, only the centre treating you will be able to view the data entered. Next, the data will be pseudonymised, i.e. your so-called personal data, e.g. name or date of birth, will be replaced by an access-protected identification code. As a result, it will no longer be possible to make a direct link between the disease-related data and your name. This will ensure that nobody else can see your data without your consent.

As you have a long-lasting illness, we will regularly examine you over a period of several years. In order to get a complete picture of the disease, we would also like to take into account, when collecting the data, information from your recent past, e.g. the first signs of your disease to become noticeable. Here, your parents will no doubt be able to help you answer these questions. This is called retrospective data collection. This data, as well as information about the future progression of your disease (known as prospective data collection), are to be collected in this data collection project.

As we are interested in collecting data from you over a very long period of time, it may be that, as you get older, you will change your doctor or move house. Even so, we would ask you to continue taking part in the data collection project and, if you and your parents agree, we would ensure that your new treating doctor receives all the information that he/she needs to take part in the data collection project.

All information to do with your disease is important to us. So, please feel free to tell your parents and doctor about anything you think is relevant.

What benefit will I gain if I take part in this data collection project?

As your current treatment is based on the doctor’s knowledge of your disease, you will probably not directly benefit from taking part in the data collection project.

By collecting data, we will learn a lot more about the disease. This may help other children with your disease. As you have a disease that may last for a very long time, it may be that you will benefit, once we know more about the disease and tell doctors what we know.

Do I have to take part in the data collection project?

It is entirely up to you to decide about taking part. Whether you do or don’t; this will not alter your medical treatment. If you change your mind over time, you should discuss this with your parents. We can then discuss together what‘s best for you.

Other important points

☑ To ensure fair play, data collection projects are reviewed before they start. This means that data collection can only start after experts from an ethics committee have given a favourable opinion. This committee also checks that the project is entirely within the law.

☑ The data collection project is being conducted out of scientific interest. However, due to the high costs associated with programming and handling data collection, this research project will firstly be backed by the pharmaceutical industry. However, the data collection project will be run independently.

☑ For data collection, a lot of data will be used. These include, for example, data on your disease and personal data.

Personal data include your name, date of birth or address. Personal data will only be used here at the clinic. For data collection, your name and date of birth will be replaced by a code number. This is known as data protection. All data will be noted down and stored on a computer. Staff at the University Hospital of Freiburg will analyse the data on behalf of the study leader. If you no longer wish to take part in the data collection project, no more data will be collected on you.

☑ Here, you will find the name of the doctor leading the data collection project, as well as the name of the doctor looking after you:

**Data collection project leader:**	**Doctor in charge at the clinic** (please enter contact details here):
Prof. Dr. Janbernd Kirschner	Name
University Hospital of Freiburg	Clinic
Department of Neuropaediatrics and Muscle Disorders	Department
	Ward
Mathildenstrasse 1	Street
79106 Freiburg i.Br.	Postcode, Town/City
Tel. +49 (0)761 270 - 43150	Tel.
Fax +49 (0)761 270 - 44750	Name and tel. no. of representative

Space for patient label

SMArtCARE
Contact person:

Consent for Participation

Data collection on disease progression in patients with spinal muscular atrophy

Project short title: SMArtCARE

Patient: ________________________________ (Surname, First name)

Date of Birth: __________________ (DD/MM/YY)

Dr. ________________________ has explained to me that this data collection project aims to find out how certain treatments work in children with the same illness as me. So, I am being asked whether I agree to take part in the data collection project.

My doctor has explained the following points to me:

- ☐ My doctor has explained the data collection project to me in detail. I have been able to ask questions about it. I have no further questions for the moment.
- ☐ I have been given information on this project. I have read this information myself/have had it read out to me by ____________________.
- ☐ I have had time to think about the data collection project and have spoken to my parents about it.
- ☐ Data collection projects are regulated by law and all laws will therefore be respected in this data collection project.
- ☐ I know that I do not have to take part in the data collection project.
- ☐ I know that my data may be collected both retrospectively and prospectively.
- ☐ I can tell the doctor or my parents at any time during the data collection project, if I no longer wish to take part. No-one will be angry with me.

I have been given the Information Sheet and will also be getting a copy of my consent.

Yes, I would like to take part in the data collection project and hereby give my name below.

________________________________ ________________

Patient's name | Date (to be filled in by the child or parents)

________________________________ ________________

Signature of the physician seeking informed consent | Date (to be filled in by the physician seeking informed consent)
