# Supplementary material for: SMArtCARE - A platform to collect real-life outcome data of patients with spinal muscular atrophy
Source: Orphanet J Rare Dis. 2019 Jan 21;14:18. doi: 10.1186/s13023-019-0998-4 (PMC6341722; doi:10.1186/s13023-019-0998-4)
Supplement: Supplementary file 3 — SMArtCARE patient information sheet for children aged 12–16 years. Consent form for the SMArtCARE data base for children aged 12–16 years. (PDF 74 kb) [file 13023_2019_998_MOESM3_ESM.pdf]

Space for patient label

SMArtCARE
Contact person:

PATIENT INFORMATION SHEET
For children and adolescents (aged 12 years and older)

Data collection on disease progression in patients with spinal muscular atrophy

Project short title: SMArtCARE

Dear Patient,

We have asked your parents or caregiver for their consent to let you take part in a data collection project in patients with spinal muscular atrophy. This will also require your approval.

Before you decide to take part in this data collection project, you should know all the main reasons why we intend to record and collect data. Before you agree to take part by signing this document, please read this information and ask anything you wish to know.

1. What is the aim of collecting data?

As you have already learned from your doctor or parents, you have a disease known as spinal muscular atrophy (SMA, for short). This disease causes muscle weakness, so that you do not always find it easy to move and you are unable to move as you would like. It can sometimes be difficult for you to breathe or cough, so you have trouble fighting off chest infections. Weakness of the muscles can cause a spine deformity known as scoliosis.

It is not yet clear exactly how best to help children with spinal muscular atrophy. There are various treatment options available, but we don't yet know which one works best. For this reason, we are collecting data from as many patients in German-speaking countries with spinal muscular atrophy as possible, in order to find out more information for better forms of treatment.

Apart from yourself, up to 1,000 patients in German-speaking countries are to take part in this data collection project. As you have an extremely rare disease, similar data collection projects are currently being set up in various countries throughout Europe. Doctors are also working together on this internationally to exchange data, in order to get a better scientific understanding on the progression of the disease. Of course, your personal data will remain secure, so that no-one will be able to link the data back to you.

2. Which data will be collected?

As part of this research project, only data documented as part of routine clinical examinations will be collected on you. If you agree, we will ask you to complete questionnaires once a year on your quality of life.

For the data collection project, we will ask you in particular about the following:

- Personal data (e.g. name, date of birth, e-mail address)
- Your age when you visit us, genetic findings, age when your symptoms started and when you were diagnosed
- Other illnesses, family history
- Current history, including your breathing status, dietary status, orthopaedic symptoms, other medications and other treatments
- Clinical examination findings, including milestones of motor development and detailed physiotherapy assessments
- Growth parameters such as height and weight, vital parameters (blood pressure, heart rate, temperature)
- Self-assessment by the patient or parents regarding changes in motor skills, breathing or dietary status and quality of life
- Information on medications specifically to treat SMA (administration, effect of treatment and tolerability)

3. What will happen in order to collect the data?

First of all, it will be checked (reviewed) whether you really have the disease described above. As this disease is rare and we don't yet know how everything fits together, this is very important. This review will be made by your doctor and would be done regardless of whether or not you take part in the data collection project. You will also have regular follow-up examinations over the next few years, but these would also be performed by your doctor, anyway. However, no extra examinations or treatments are scheduled. You will always be treated to the best of your doctor's ability.

If you and your parents agree to take part in the project, your data will be entered into a database. To begin with, only the centre treating you will be able to view the data entered. Next, the data will be pseudonymised, by replacing your so-called personal data, e.g. name or date of birth, by an access-protected identification code. As a result, it will no longer be possible to make a direct link between the disease-related data and your name. This will ensure that nobody else can see your data without your consent.

As you have a long-lasting illness, you will be regularly interviewed and examined over a period of several years. In order to get a complete picture of the disease, we would also like to take into account, in the registry, information that has come to light since you first developed the disease. This is called retrospective data collection. This data, as well as information about the future progression of your disease (known as prospective data collection), are to be collected in this data collection project.

4. What will happen if I change my doctor?

As we are interested in collecting data from you over a very long period of time, it may be that, as you get older, you will change your doctor or move house. Even so, we would ask you to continue taking part in the data collection project and, if you and your parents agree, we would ensure that your new treating doctor receives all the information that he/she needs to take part in the data collection project.

5. How will I benefit from taking part?

As we wish to learn a lot more about your disease with this data collection project, you will probably not directly benefit by taking part.

However, your willingness may possibly help other children and adolescents with the same disease. As you have a chronic disease, i.e. a long-term illness lasting over many years, it is possible that you still might benefit from what we find out at a later date.

6. Do I have to take part in the data collection project?

It is entirely up to you whether or not you decide to take part. Even at a later date, you can still withdraw your consent; no-one will hold this against you. If you wish to withdraw your participation before the end of the project, please always let your doctor know. He/she will want to make sure that everything is OK.

7. What will happen to my collected data?

For data analysis, a lot of data will be used, mainly data about your disease and your examinations. However, your full name or exact date of birth will not be recorded at any point. These personal data will be encrypted, i.e. replaced by a code number.

Apart from your doctor, only very specific people may access this personal data. These include, for example, colleagues of the data collection project leader. However, these persons will not be allowed to disclose any of this information. This is known as confidentiality. Your data will be treated in absolute confidence and will not be made public.

The data will be used by doctors and scientists around the world to better understand your disease. It is not just doctors working in hospitals who will be working on the data, but also scientists in the pharmaceutical industry. Transfer of your data will be decided by the leader of this data collection project, together with an independent committee.

8. What else should I know?

Data collection will be coordinated by a study leader. You can contact him and, of course, your treating doctor at any time. They will be happy to answer any questions you may have.

**Data collection project leader:**	**Doctor in charge at the clinic** (please enter contact details here):
Prof. Dr. Janbernd Kirschner	Name
University Hospital of Freiburg	Clinic
Department of Neuropaediatrics and Muscle Disorders	Department
Mathildenstrasse 1	Ward
79106 Freiburg i.Br.	Street
	Postcode, Town/City
Tel. +49 (0)761 270-43150	Tel.
Fax +49 (0)761 270-44750	Name and tel. no. of representative

Space for patient label

SMArtCARE

Contact person:

Consent for Participation

Data collection on disease progression in patients with spinal muscular atrophy

Project short title: SMArtCARE

Patient: ______________________________ Date of Birth: ________________

(Surname, First name) (DD/MM/YY)

- ☐ I have read the Patient Information Sheet. I have understood what this information contains.
- ☐ My doctor has given me an explanation of the scheduled data collection project in a detailed personal discussion.
- ☐ I have had the opportunity to ask questions and have had time to decide about taking part in the registry. I have no further questions for the moment. My doctor will remain at my disposal at any time for any queries.
- ☐ I have been told that my personal information will be treated confidentially. When data about my disease are collected and evaluated, it will not be possible to trace them back to me personally. All persons having access to my personal data will be obliged to maintain confidentiality towards third parties.
- ☐ I know that participation in the data collection project is voluntary. My parents have given their consent for participation and I also agree. I know that it is up to me to decide whether to give my consent.
- ☐ I know that my data may be collected both retrospectively and prospectively.
- ☐ I agree to quality-of-life questionnaires being sent to the e-mail address provided by me

  ☐ Yes

  ☐ No
- ☐ Optional: If I should change my doctor, I agree to my data being made available to my new treating doctor.

  ☐ Yes

  ☐ No
- ☐ I have been told that I can withdraw my participation in the data collection project at any time. If I should withdraw my participation, I will let my doctor know.
- ☐ I have been given a copy of this Informed Consent Form and Patient Information Sheet.

By entering today's date and giving my signature below, I hereby agree to take part in the data collection project.

______________________________ ________________ ______________________________

Patient Date* Signature

By giving his/her signature, the physician seeking consent hereby confirms that he/she has conducted the informed consent discussion and has obtained the patient's consent.

______________________________ ________________ ______________________________

Name of the physician seeking informed consent Date* Signature

* The date must be entered by each person by hand.
