# Supplementary material for: SMArtCARE - A platform to collect real-life outcome data of patients with spinal muscular atrophy
Source: Orphanet J Rare Dis. 2019 Jan 21;14:18. doi: 10.1186/s13023-019-0998-4 (PMC6341722; doi:10.1186/s13023-019-0998-4)
Supplement: Supplementary file 4 — SMArtCARE parent information sheet. Consent form for the SMArtCARE data base for parents. (PDF 106 kb) [file 13023_2019_998_MOESM4_ESM.pdf]

Space for patient label

SMArtCARE
Contact person:

PARENT INFORMATION SHEET

Data collection on disease progression in patients with spinal muscular atrophy

Project short title: SMArtCARE

Dear Parents / Legal Representative,

With this Information Sheet, we would like to inform you and your child, a sufferer of spinal muscular atrophy, about the possibility of taking part in a data collection project called "SMArtCARE". This is a scientific research project, run by the Department of Neuropaediatrics and Muscle Disorders at the University Hospital of Freiburg.

This written Information Sheet is being issued in conjunction with a verbal explanation and aims to provide you with key information, so that you - if applicable, together with your child - can decide on the participation of your child in this research project. Your child's participation is voluntary. Before you decide whether to give your consent for participation, it is important that you fully understand the nature of data collection and what it involves, and that you have been informed of your rights and obligations. Please do not hesitate to ask for some time to make up your mind, if you need it. Your decision about taking part in the data collection project will not affect your child's treatment in any way.

1. Why is this research project being performed?

Your child has been diagnosed with a rare disease known as spinal muscular atrophy (muscle wastage, SMA). For the first time, the natural progression of the disease can be positively influenced by new drug therapies. The SMArtCARE data collection project aims to collect data from as many patients as possible with SMA, in order to better understand the progression of the disease and thus improve the treatment of patients with SMA. To this end, we also intend to enter your child's data, collected by us during treatment, in this database and analyse it together with data from other patients.

In addition to your child's data, it is planned to collect data from more than 1,000 other patients in German-speaking countries. As SMA is a rare disease, similar data collection projects are currently being set up in various countries throughout Europe. Doctors are also working together on this internationally to exchange data, in order to get a better scientific understanding on the progression of the disease. Of course, your child's personal data will remain secure, so that no-one will be able to link the data back to your child.

This data collection project is being conducted out of scientific interest. However, due to the high costs associated with programming and handling data collection, this research project will initially be backed by the pharmaceutical industry. However, the data collection project will be run independently from the pharmaceutical industry.

2. Which data will be recorded?

As part of this research project, only data documented as part of routine clinical examinations will be collected on your child. Provided you agree, you will also be asked to complete questionnaires once a year on your child's quality of life.

More specifically, the following data will be recorded at the first visit and during the project:

- Personal data (e.g. name, date of birth, e-mail address)
- Age at the time of visit, genetic findings, age when symptoms started and at diagnosis
- Other illnesses, family history
- Current history, including breathing status, dietary status, orthopaedic symptoms, other medications and other treatments
- Clinical examination findings, including milestones of motor development and detailed physiotherapy assessments
- Growth parameters such as height and weight, vital parameters (blood pressure, heart rate, temperature)
- Self-assessment by the patient or parents regarding changes in motor skills, breathing or dietary status and quality of life
- Information on medications specifically to treat SMA (administration, effect of treatment and tolerability)

3. How will this project unfold?

Your child is being treated in accordance with the currently best known standards and recommendations for SMA. With your consent, your child's data will be entered into a database. To begin with, only the centre treating your child will be able to view your child's entered data. Next, the data will be pseudonymised, by replacing your child's personal data (e.g. name or date of birth) by an access-protected identification code, so that no direct link can be made between the disease-related data and your child's name. This will ensure that no third parties will be able to see these data without your consent.

In order to get a complete picture on the progression of your child's disease, we would also like to take into account, when collecting the data, information that has come to light since your child first developed the disease. This is called retrospective data collection. This data, as well as information about the future progression of the disease (known as prospective data collection), are to be collected in this data collection project.

4. Optional: What will happen if I change my treating doctor?

As we are interested in collecting data from your child over a long period of time, you may meanwhile change your doctor or move house. Even so, we would ask your child to continue taking part in the data collection project and, provided you agree, we would ensure that the new treating doctor receives all the information that he/she needs to take part in the data collection project.

5. What personal benefit or risks are there for your child as a result of taking part in the research project?

Your child will probably not gain any immediate personal health benefit just by taking part in this research project. However, the research findings may possibly help improve the future treatment of this disease. As the disease is chronic, your child may benefit in future just from the experience alone gained from this research.

6. Can consent for participation in the project be withdrawn at any time?

Participation in the research project is voluntary and requires your written consent. You can withdraw participation at any time without giving any reasons. If you should wish to withdraw your participation, please contact your treating doctor. In this case, no more new data would be recorded. As the data collection project is designed to last for a very long time and evaluations and publications are to be carried out at regular intervals, data already evaluated and published can no longer be deleted. However, these data will be anonymised by deleting your child's personal data (e.g. name or date of birth), so that a link can no longer be made between your child and his/her data. By withdrawing your consent, neither you or nor your child will be disadvantaged regarding further treatment, nor will you be waiving any rights to which you would otherwise be entitled. The relationship of trust between you and your doctor will likewise not be harmed. Please tell your doctor if you wish to end your child's participation in the research project.

7. What will happen to the collected data?

Your child's data will be processed and analysed in a database run by staff at the University Hospital of Freiburg. The conditions of medical confidentiality and data protection will be respected. The database used in this research project has its own independent data protection concept.

The data will be evaluated within the framework of research projects by national and international scientists conducting research into the indication of SMA, both in a clinical and pharmaceutical/industrial environment. The leader in charge of the research project (Prof. Dr. Janbernd Kirschner) will decide how these data will be made available after consultation and consensus with an independent steering committee. In the event of any publication of research findings referring to your child's data, it will be ensured that patient identity will not be revealed (anonymised). Transfer of data to unauthorised third parties (particularly employers, insurance companies or investigating authorities) is excluded.

You will have the right to inspect your child's data and to amend any incorrect details.

With regard to all data stored in the database, personal data will be deleted no later than 10 years after the end of data collection, so that no link to the identity of patients can be established (anonymised).

8. Whom can you contact if you have any further questions?

If you still have any further questions, please contact your treating doctor or the doctor in charge of the research project and data processing:

**Data collection project leader:**	**Doctor in charge at the clinic** (please enter contact details here):
Prof. Dr. Janbernd Kirschner	Name
University Hospital of Freiburg	Clinic
Department of Neuropaediatrics and Muscle Disorders	Department
	Ward
Mathildenstrasse 1	Street
79106 Freiburg i.Br.	Postcode, Town/City
Tel. +49 (0)761 270-43150	Tel.
Fax +49 (0)761 270-44750	Name and tel. no. of representative

For questions on data protection, you can also contact the following agent(s):

**Relevant data protection officer in the project management team:**	**Relevant local data protection officer:** (please enter contact details here):
datenschutz@uniklinik-freiburg.de	e-mail
University Hospital of Freiburg	Clinic
Data protection officer	Department
Agnesenstrasse 6 – 8	Street
79106 Freiburg	Postcode, Town/City

Right to lodge a complaint

You have the right to lodge a complaint. If you believe that processing of your child's personal data contravenes the European General Data Protection Regulation (GDPR) (Article 77 EUR-GDPR), please contact the lead supervisory authority responsible for the University Hospital of Freiburg (*UKF*):

Federal Commissioner for Data Protection and Freedom of Information

Street address	Postal address
Königstrasse 10 a	Postfach<br>10 29 32 (PO Box)
70173 Stuttgart	70025 Stuttgart
tel.: +49 (0)711/61 55 41 – 0	
fax: +49 (0)711/61 55 41 – 15	
e-mail: poststelle@lfdi.bwl.de	
Internet: http://www.baden-wuerttemberg.datenschutz.de	

Space for patient label

SMArtCARE
Contact person:

Informed Consent Form

Data collection on disease progression in patients with spinal muscular atrophy

Project short title: SMArtCARE

Patient: ______________________________ (Surname, First name)

Date of Birth: ________________ (DD/MM/YY)

We have been sufficiently informed, both verbally and in writing, about the planned scientific research project. All questions raised by us about this project have been answered satisfactorily. By giving our handwritten signature, we hereby confirm our consent to the voluntary participation of our child in this data collection project. We declare that we give our consent taking into account the will of our child and we confirm by signing that this is the case.

- We have been told that our child's data will be transferred to scientists, who will use them for research projects aimed at better understanding the progression of SMA
- We have been told that our child's data will be collected both retrospectively and prospectively;
- We have been told that we can withdraw our consent at any time without giving any reasons. Similarly, our child can also withdraw his/her participation in the data collection project at any time, without any prejudice to his/her further medical care. In the event of withdrawal, we hereby agree that the data collected up until that time may continue to be used for the research project, after deletion of personal data.
- We agree to quality-of-life questionnaires being sent to the e-mail address provided by us.

☐ Yes
☐ No

- Optional: If we should change our treating doctor, we agree to our child's data being made available to the new treating doctor.

☐ Yes
☐ No

Data Protection - Information and Consent:

We declare that we have been informed that, as part of the project, our child's personal data will be recorded in encrypted form, stored and evaluated at the University Hospital of Freiburg, and we agree to the above.

The legal basis for the processing of personal data is your consent in accordance with the purpose of this research project (described in section 1), pursuant to Art. 9 (2a) of the EU-GDPR.

I am aware that persons duly authorised by the leader/deputy of this data collection project may inspect my child's medical records for control purposes. I agree to such inspection, which is permitted only in relation to this data collection study.

In the event of data transfer to collaboration partners, or use of the data for scientific purposes or for publication, it will not possible to trace the data back to our child's identity. I am aware that, in some countries (e.g. USA), the standard of data protection is lower than in the EU.

We have been informed that we

- have the right to demand information on our child's personal data (Article 15 EU-GDPR) and to receive a copy of these data free of charge.
- have the right to demand the rectification (correction) of our child's personal data, if these should prove to be incomplete or inaccurate (Article 16 EU-GDPR).
- have a right to erase our child's personal data (Article 17 EU-GDPR). However, this right is not absolute. The right to data erasure does not apply if invocation of this right would seriously affect the correct scientific implementation of the research project or render it impossible (Art. 17 (3d) EU-GDPR).

On the basis of pending legislation by the State of Baden-Württemberg with regard to processing of our child's data at the University Hospital Freiburg, our rights to information, rectification and erasure (see above) may be restricted in time and/or in terms of content, inasmuch as this is required to avoid the probability that the correct scientific implementation of the research project would be seriously affected or rendered impossible.

We have been informed that we have a right to lodge complaints (see section 8).

We have been informed that we have the option of withdrawing our consent for the processing of our child's personal data at any time, without giving reasons (Art. 7 (3) EU-GDPR, see section 6) and without affecting the lawfulness of processing our child's personal data up until withdrawal of said consent. In the event of any withdrawal of participation in the study, registry data already pseudonymised will then be anonymised (= made no longer traceable). Data sets cannot be deleted but will be anonymised. Registry data from evaluation data sets already created will not be anonymised.

We hereby agree to the storage of our child's data for ten years after completion or termination of the study. Thereafter, the personal data will be anonymised, unless legally binding retention periods dictate otherwise.

We have been given a copy of this Patient Information Sheet and Informed Consent Form. As the research project will last for a number of years, we have been recommended to keep these documents for any questions at a later date.

Parent / Custodian	Date*	Signature
Parent / Custodian	Date*	Signature

By giving his/her signature, the physician seeking consent hereby confirms that he/she has conducted the informed consent discussion and has obtained the patient’s consent.

Name of the physician seeking informed consent	Date*	Signature

* The date must be entered by each person by hand.
