# Supplementary material for: SMArtCARE - A platform to collect real-life outcome data of patients with spinal muscular atrophy
Source: Orphanet J Rare Dis. 2019 Jan 21;14:18. doi: 10.1186/s13023-019-0998-4 (PMC6341722; doi:10.1186/s13023-019-0998-4)
Supplement: Supplementary file 5 — SMArtCARE patient information sheet. Consent form for the SMArtCARE data base for patients. (PDF 104 kb) [file 13023_2019_998_MOESM5_ESM.pdf]

Space for patient label

**SMArtCARE**
**Contact person:**

# PATIENT INFORMATION SHEET
## Data collection on disease progression in patients with spinal muscular atrophy
Project short title: SMArtCARE

Dear Patient,

You are being treated at our centre because you have been diagnosed with muscular atrophy (muscle wastage). In this respect, we would like to inform you, with this Information Sheet, about the possibility of taking part in a data collection project called “SMArtCARE”. This is a scientific research project, run by the Department of Neuropaediatrics and Muscle Disorders at the University Hospital of Freiburg.

This written Information Sheet is being issued in conjunction with a verbal explanation and aims to provide you with key information, so that you can decide on voluntary participation in this research project. Before you decide whether to give your consent for participation, it is important that you fully understand the nature of data collection and what it involves, and you have been informed of your rights and obligations. Please do not hesitate to ask for some time to make up your mind, if you need it. Your decision about taking part in the data collection project will not affect your treatment in any way.

### 1. Why is this research project being performed?

You have been diagnosed with a very rare disease known as spinal muscular atrophy (SMA). For the first time, the natural progression of the disease can be positively influenced by new drug therapies. The SMArtCARE data collection project aims to collect data from as many patients as possible with SMA, in order to better understand the progression of the disease and thus improve the treatment of patients with SMA. To this end, we also intend to enter your data, collected by us during treatment, in this database and analyse it together with data from other patients.

In addition to your data, it is planned to collect data from more than 1,000 other patients in German-speaking countries. As SMA is a rare disease, similar data collection projects are currently being set up in various countries throughout Europe. Doctors are also working together on this internationally to exchange data, in order to get a better scientific understanding on the progression of the disease. Of course, your personal data will remain secure, so that no-one will be able to link the data back to you.

This data collection project is being conducted out of scientific interest. However, due to the high costs associated with programming and handling data collection, this research project will initially be backed by the pharmaceutical industry. However, the data collection project will be run independently from the pharmaceutical industry.

## 2. Which data will be recorded?

As part of this research project, only data documented as part of routine clinical examinations will be collected on you. Provided you agree, you will also be asked to complete questionnaires once a year on your quality of life.

More specifically, the following data will be recorded at the first visit and during the project:

- Personal data (e.g. name, date of birth, e-mail address)
- Age at the time of visit, genetic findings, age when symptoms started and at diagnosis
- Other illnesses, family history
- Current history, including breathing status, dietary status, orthopaedic symptoms, other medications and other treatments
- Clinical examination findings, including milestones of motor development and detailed physiotherapy assessments
- Growth parameters such as height and weight, vital parameters (blood pressure, heart rate, temperature)
- Self-assessment by the patient or parents regarding changes in motor skills, breathing or dietary status and quality of life
- Information on medications specifically to treat SMA (administration, effect of treatment and tolerability)

## 3. How will this project unfold?

You are being treated in accordance with the currently best known standards and recommendations for SMA.

If you decide to take part in the data collection project, your data will be entered into a database. To begin with, only the centre treating you will be able to view your entered data. Next, the data will be pseudonymised, by replacing your personal data (e.g. name or date of birth) by an access-protected identification code, so that no direct link can be made between the disease-related data and your name. This will ensure that no third parties will be able to see these data without your consent.

As part of the data collection project, only data relating to your established therapy at your centre will be recorded. You have already been informed about this separately. No additional examinations will take place. In order to get a complete picture on the progression of your disease, we would also like to take into account, when collecting the data, information that has come to light since you first developed the disease. This is called retrospective data collection. This data, as well as information about the future progression of the disease (known as prospective data collection), are to be collected.

## 4. Optional: What will happen if I change my treating doctor?

As we are interested in collecting data on you over a long period of time, you may meanwhile change your doctor or move house. Even so, we would ask you to continue taking part in the data collection project and, provided you agree, we would ensure that the new treating doctor receives all the information that he/she needs to take part in the data collection project.

## 5. What personal benefit or risks are there for you as a result of taking part in the research project?

You will probably not gain any immediate personal health benefit just by taking part in this research project. However, the research findings may possibly help find ways of treating this disease in the future. As the disease is chronic, you may benefit in future just from the experience alone gained from this research.

## 6. Can consent for participation in the project be withdrawn at any time?

Participation in the research project is voluntary and requires your written consent. You can withdraw participation at any time without giving any reasons. If you should wish to withdraw your participation, please contact your treating doctor. In this case, no more new data would be recorded. As the data collection project is designed to last for a very long time and evaluations and publications are to be carried out at regular intervals, data already evaluated and published can no longer be deleted. However, these data will be anonymised by deleting your personal data (e.g. name or date of birth), so that a link can no longer be made between you and your data.

By withdrawing your consent, you will be disadvantaged regarding further treatment, nor will you be waiving any rights to which you would otherwise be entitled. The relationship of trust between you and your doctor will likewise not be harmed. Please tell your doctor if you wish to end your participation in the research project.

## 7. What will happen to the collected data?

Your data will be processed and analysed in a database run by staff at the University Hospital of Freiburg. The conditions of medical confidentiality and data protection will be respected. The database used in this research project has its own independent data protection concept.

The data will be evaluated within the framework of research projects by national and international scientists conducting research into the indication of SMA, both in a clinical and pharmaceutical/industrial environment. The leader in charge of the research project (Prof. Dr. Janbernd Kirschner) will decide how these data will be made available after consultation and consensus with an independent steering committee. In the event of any publication of research findings referring to your data, it will be ensured that patient identity will not be revealed (anonymised). Transfer of data to unauthorised third parties (particularly employers, insurance companies or investigating authorities) is excluded.

You will have the right to inspect your data and to amend any incorrect details.

With regard to all data stored in the database, personal data will be deleted no later than 10 years after the end of data collection, so that no link to the identity of patients can be established (anonymised).

## 8. Whom can you contact if you have any further questions?

If you still have any further questions, please contact your treating doctor or the doctor in charge of the research project and data processing:

| **Data collection project leader:** | **Doctor in charge at the clinic:** (please enter contact details here): |
|---|---|
| Prof. Dr. Janbernd Kirschner | Name |
| University Hospital of Freiburg | Clinic |
| Department of Neuropaediatrics and Muscle Disorders | Department |
| | Ward |
| Mathildenstrasse 1 | Street |
| 79106 Freiburg i.Br. | Postcode, Town/City |
| Tel. +49 (0)761 270-43150 | Tel. |
| Fax +49 (0)761 270-44750 | Name and tel. no. of representative |

For questions on data protection, you can also contact the following agent(s):

| **Relevant data protection officer in the project management team:** | **Relevant local data protection officer:** (please enter contact details here): |
|---|---|
| datenschutz@uniklinik-freiburg.de | E-mail |
| University Hospital of Freiburg | Clinic |
| Data protection officer | Department |
| Agnesenstrasse 6-8 | Street |
| 79106 Freiburg | Postcode, Town/City |

### Right to lodge a complaint

You have the right to lodge a complaint. If you believe that processing of your personal data contravenes the European General Data Protection Regulation (GDPR) (Article 77 EUR-GDPR), please contact the lead supervisory authority responsible for the University Hospital of Freiburg (*UKF*):

**Federal Commissioner for Data Protection and Freedom of Information**

| Street address | Postal address |
| --- | --- |
| Königstrasse 10 a | Postfach<br>10 29 32 (PO Box) |
| 70173 Stuttgart | 70025 Stuttgart |
| tel.: +49 (0)711/61 55 41 – 0 | |
| fax: +49 (0)711/61 55 41 – 15 | |
| e-mail: poststelle@lfdi.bwl.de | |
| Internet: http://www.baden-wuerttemberg.datenschutz.de | |

Space for patient label

**SMArtCARE**

**Contact person:**

# Informed Consent Form

## Data collection on disease progression in patients with spinal muscular atrophy

Project short title: SMArtCARE

**Patient:**________________________________ **Date of Birth:** ________________
(Name, First name) (DD/MM/YY)

I have received detailed information on the above-mentioned data collection project for the recording and analysis of patient data. I have had sufficient opportunity to ask all my questions. These have been answered completely and satisfactorily. I have received, read and understood the written Patient Information Sheet on the above-mentioned data collection project. I have had sufficient time to make up my mind.

I have been informed that my participation is voluntary and that I have the right to end it at any time, without giving any reasons and without prejudice to myself.

- I have been told that my data will be transferred to scientists, who will use them for research projects aimed at better understanding the progression of SMA
- I have been told that my data will be collected both retrospectively and prospectively
- I have been told that I can withdraw my consent at any time without giving any reasons and without any prejudice to my further medical care. In the event of withdrawal, I hereby agree that the data collected up until that time may continue to be used for the research project (anonymised), after deletion of personal data.
- I agree to quality-of-life questionnaires being sent to the e-mail address provided by me.

☐ Yes

☐ No

- Optional: If I should change my doctor, I agree to my data being made available to my new treating doctor.

☐ Yes

☐ No

**<u>Data Protection - Information and Consent:</u>**

**I hereby agree to take part in the data collection project and agree that, as part of the project, my personal data will be recorded in encrypted form, stored and evaluated at the University Hospital of Freiburg.**

The legal basis for the processing of personal data is your consent in accordance with the purpose of this research project (described in section 1), pursuant to Art. 9 (2a) of the EU-GDPR.

I am aware that persons duly authorised by the leader/deputy of this data collection project may inspect my medical records for control purposes. I agree to such inspection, which is permitted only in relation to this data collection study.

In the event of data transfer to collaboration partners, or use of the data for scientific purposes or for publication, it will not possible to trace the data back to my identity. I am aware that, in some countries (e.g. USA), the standard of data protection is lower than in the EU.

I have been informed that I

- have the right to demand information on my personal data (Article 15 EU-GDPR) and to receive a copy of these data free of charge.

- have the right to demand the rectification (correction) of my personal data, if these should prove to be incomplete or inaccurate (Article 16 EU-GDPR).
- have a right to erase my personal data (Article 17 EU-GDPR). However, this right is not absolute. The right to data erasure does not apply if invocation of this right would seriously affect the correct scientific implementation of the research project or render it impossible (Art. 17 (3d) EU-GDPR).

On the basis of pending legislation by the State of Baden-Württemberg with regard to processing of my data at the University Hospital Freiburg, my rights to information, rectification and erasure (see above) may be restricted in time and/or in terms of content, inasmuch as this is required to avoid the probability that the correct scientific implementation of the research project would be seriously affected or rendered impossible.

I have been informed that I have a right to lodge complaints (see section 8).

I have been informed that I have the option of withdrawing my consent for the processing of my personal data at any time, without giving reasons (<u>Art. 7 (3) EU-GDPR, see section 6)</u> and without affecting the lawfulness of processing my personal data up until withdrawal of said consent. In the event of any withdrawal of participation in the study, registry data already pseudonymised will then be anonymised (= made no longer traceable). Data sets cannot be deleted but will be anonymised. Registry data from evaluation data sets already created will not be anonymised.

I hereby agree to the storage of my data for ten years after completion or termination of the study. Thereafter, my personal data will be anonymised, unless legally binding retention periods dictate otherwise.

I have been given a copy of this Patient Information Sheet and Informed Consent Form. As the research project will last for a number of years, I have been recommended to keep these documents for any questions at a later date.

| | | |
|---|---|---|
| Patient | Date* | Signature |

By giving his/her signature, the physician seeking consent hereby confirms that he/she has conducted the informed consent discussion and has obtained the patient's consent.

| | | |
|---|---|---|
| Name of the physician<br>seeking informed consent | Date* | Signature |

* The date must be entered by each person by hand.
